# MPO/HOCl Facilitates Apoptosis and Ferroptosis in the SOD1^G93A^ Motor Neuron of Amyotrophic Lateral Sclerosis

**DOI:** 10.1155/2022/8217663

**Published:** 2022-02-07

**Authors:** Jialing Peng, Jingrui Pan, Jingjing Mo, Ying Peng

**Affiliations:** ^1^Department of Neurology, Sun Yat-sen Memorial Hospital, Sun Yat-sen University, Guangzhou, China; ^2^Guangdong Provincial Key Laboratory of Malignant Tumor Epigenetics and Gene Regulation, Sun Yat-sen Memorial Hospital, Sun Yat-sen University, Guangzhou, China

## Abstract

**Background:**

Oxidative stress and reactive oxygen species (ROS) are important in the pathogenesis of amyotrophic lateral sclerosis (ALS). Hypochlorous acid (HOCl) is a powerful oxidant of the reactive oxygen species (ROS) family. HOCl's role in the progress of ALS remains unclear due to the lack of an effective HOCl detection method. Cumulative evidence supports oxidative damage incurred by mutant hSOD1 contributing to motor neuron death; however, whether HOCl as well as its catalytic enzyme myeloperoxidase (MPO) function in the cell death of SOD1^G93A^ ALS remains elusive.

**Methods:**

The hSOD1^WT^ and hSOD1^G93A^ NSC-34 cell and SOD1^G93A^ ALS mouse models were employed. With a novel fluorescent HOCl probe, HKOCl-3, we detected the expressions of HOCl and its catalytic enzyme, MPO, in the above models *in vitro* and *in vivo*. The regulation of MPO/HOCl by hSOD1^G93A^ mutation and cell deaths by MPO/HOCl were also assayed, including apoptosis, ferroptosis, and autophagy.

**Results:**

Our results showed that hSOD1^G93A^ mutation promoted the activation of the MPO/HOCl pathway in SOD1^G93A^ ALS cell models. The activation of MPO/HOCl pathways facilitated apoptosis and ferroptosis through increasing the Bax/Bcl-2 ratio and expression of caspase-3 or inhibiting the expressions of GPX4 and NQO1 and thus leading to irreversible lipid peroxidation. Overexpressed FSP1, a glutathione-independent suppressor, could ameliorate ferroptosis. *In vivo*, we demonstrated that the activation of the MPO/HOCl pathway occurred differently in motor neurons of the motor cortices, brain stems, and spinal cords in male and female SOD1^G93A^ transgenic mice. In addition, inhibiting MPO improved the motor performance of SOD1^G93A^ transgenic mice, as demonstrated by the rotarod test.

**Conclusions:**

We concluded that aggregation of mutant hSOD1 proteins contributed to activation of the MPO/HOCl pathway, triggering apoptosis and ferroptosis in motor neuronal deaths and exerting impaired motor performance.

## 1. Introduction

Amyotrophic lateral sclerosis (ALS), a progressive and fatal neurodegenerative disease, is characterized by selective neuronal deaths in the spinal cord, brain stem, and motor cortex. Due to a lack of effective treatments, patients die within 3-5 years after symptom onset [1]. About 10% of ALS cases are caused by identified mutant genes with inherited forms, such as chromosome 9 open reading frame 72 (*C9orf72*), copper/zinc superoxide dismutase 1 (*SOD1*) [2], and TARDNA-binding protein (*TARDBP*) [3], among which SOD1 is reported to be the most prevalent in Asian ALS patients (30%) [4]. Misfolded SOD1 proteins are failed to be identified by an impaired ubiquitin proteasome system and degradation, thus leading to insoluble protein aggregation [5]. Then, oxidative damage caused by SOD1 aggregation is highlighted, including protein and lipid peroxidation to induce irreversible motor neuronal death [6]. Proposedly, misfolded SOD1 proteins are deposited on organelles, especially mitochondria, and impair mitochondrial function [7–9], and then, the dysfunctional mitochondria cause cellular ROS accumulation, serving as a key mediator in a vicious cycle of oxidative stress and exacerbated mitochondrial dysfunction [10]. Accumulated reactive oxygen species (ROS), such as hydrogen peroxide and hydroxyl free radicals, play a critical role in mediating oxidative stress in ALS, resulting in lipid peroxidation and genotoxicity [11].

Ferroptosis was coined as a nonapoptotic form of cell death in 2012 [12] and has been well-characterized by lethal lipid peroxidation due to failure of antioxidant defenses and the excess of ROS. The value of the neuronal susceptibility of ferroptosis triggered by oxidative stress has been demonstrated in previous studies [13]. Nonetheless, little is known about whether and how ferroptosis contributes to ALS. Ferroptosis has been suggested as the nexus of redox biology and cellular health, sharing some common pathways with other forms of cell death, such as apoptosis and autophagy [14, 15]. Although lipid peroxidation has been recognized as a signature of ferroptosis, the products of lipid peroxidation were confirmed to initiate apoptosis by MAPK (mitogen-activated protein kinases) [16], PKC (protein kinase C) [17] pathways, and so on. The apoptotic inducer, p53 protein, was demonstrated to disturb the elimination of ROS and lipid peroxide, facilitating the switching of apoptosis to ferroptosis [18]. Thus, it is conceivable that ferroptosis might affect the fate of cell in an integrative way, together with apoptosis or autophagy.

Hypochlorous acid (HOCl) is a member of the ROS family and a powerful reactive oxygen intermediate, which is generated from catalyzation by a heme-containing enzyme, myeloperoxidase (MPO). MPO converts hydrogen peroxide (H_2_O_2_) and Cl^−^ to H_2_O to form HOCl [19], gaining increased attention for their oxidative ability and contribution to cytotoxicity in inflammatory diseases. MPO has been proposed as a biomarker and important therapeutic target in various neurodegenerative diseases, including multiple sclerosis, Alzheimer's disease, and Parkinson's disease [20, 21]. However, the cellular function of HOCl, the precursor of free radicals and predominant neurotoxic oxidant in the brain [22], during oxidative damage remains poorly understood due to the lack of a sensitive HOCl detection, impeding exploration of the mechanisms of MPO/HOCl during pathologic processes. We utilized a novel and selective HOCl fluorescent probe, HKOCl-3, to uncover the pathogenesis of MPO/HOCl in an ALS model of SOD1^G93A^, a form of *SOD1* mutation with the alteration at the 93rd codon from glycine to alanine [23]. We confirmed the regulation of MPO/HOCl in lipid peroxidation and thus induced ferroptosis and apoptosis in the neuron of the ALS model. Eventually, activation of MPO/HOCl potentiated neuronal death and neurological deficits.

## 2. Methods

### 2.1. Reagents and Plasmids

4-Aminobenzoic acid hydrazide (ABAH, Cayman, CAC-14845-1), ferrostatin-1 (MCE, HY-100579), Z-DEVD-FMK (MCE, HY-12466), and puromycin (Sigma-Aldrich, P7255) were used. The following plasmids were used: *hSOD1* empty plasmid (EX-NEG-Lv201), GFP-*hSOD1^WT^* (EX-K2710-Lv201), GFP-*hSOD1^G93A^* (CS-K2710-Lv201), *FSP1* empty plasmid (EX-NEG-Lv105), *FSP1* (EX-Mm35867-Lv105), *MPO* empty plasmid (EX-NEG-Lv206), and mCherry-*MPO* (EX-Mm34293-Lv206) were purchased from GeneCopoeia. Packaging plasmids including psPAX2 and pMD2.G were used to generate lentivirus.

### 2.2. Cell Culture and Transfection

The NSC-34 cell line was obtained from American Type Culture Collection and cultured in complete DMEM (HyClone, SH30243.01) which contains 10% fetal bovine serum (FBS) (Gibco, 10099–141) and 1% penicillin/streptomycin (ThermoFisher, 15140122) at 37°C and 5% CO_2_. For transient transfection, NSC-34 cells were transfected with empty plasmid (GFP-vector), GFP-*hSOD1^WT^* plasmid, and GFP-*hSOD1^G93A^* plasmid using Lipofectamine 3000 reagents (Invitrogen, L3000001). 36 h after transfection, the GFP-positive cells were sorted by a flow cytometer (LSR II, BD) and then cultured again. Lentivirus was generated in 293 Ft cells according to the manufacturer's instruction of Lipofectamine 3000 reagents, and puromycin was used for stably transfected cells. For siRNA transfection, siRNAs were obtained from RiboBio (Guangzhou, China). The siRNA were used as follows: mouse NC-siRNA, mouse MPO-siRNA (siG170907021928), human NC-siRNA, and hSOD1-siRNA (stB0003856A).

### 2.3. Volunteer Enrollment and Animal Model

We enrolled 26 aged-matched and gender-matched healthy volunteers and ALS patients from Sun Yat-sen Memorial Hospital, Sun Yat-sen University. The trial was registered in the Chinese Clinical Trial Register (ChiCTR1900023321) and approved by the Medical Ethic Committee of Sun Yat-sen Memorial Hospital (SYSEC-KY-KS-2019-014). We took informed consent of the volunteers. The blood samples were obtained from all the volunteers, and ALS patients were evaluated by the ALS Functional Rating Scale-Revised (ALSFRS) score. Fresh blood samples once collected were centrifuged at 2,000 rpm for 20 min; then, the plasma was collected and stored at −80°C until used, in order to avoid degradation of HOCl at room temperature *in vitro*.

Transgenic mice carrying the human *SOD1* (*hSOD1^WT^*) gene and human *SOD1^G93A^* (*hSOD1^G93A^*) gene were bred as previous studies suggested [24, 25]. Mice were maintained by a pure C57BL6 background and genotyped by gel electrophoresis. Animals were free for water and food and housed in cages with a 12 h light/dark cycle. Experimental procedures were approved by the Institutional Animal Care and Use Committee, Sun Yat-sen University (SYSU-IACUC-2019-000026). According to previous studies [26, 27], *hSOD1^G93A^* high copy mice were balanced for sex and age including preonset (60 d), early stage (90 d), and late stage (120 d). Age- and sex-matched *hSOD1^WT^* mice served as controls. 100 mg/kg ABAH was injected intraperitoneally twice/day for 7 days.

### 2.4. Detection of HOCl Levels by HKOCl-3 Fluorescent Probe

The fluorescent signal of the modified HKOCl-3 probe [28] could be detected independently in both green and red ranges. Supernatants of live cells were collected, and cells were washed by PBS and replaced with complete medium contained with 10 *μ*M HKOCl-3. Incubation was performed at 37°C in the dark for 30 min, and images were captured by fluorescent microscopy (Olympus lx71, Japan). The intensities of red fluorescence were analyzed by ImageJ to quantify the levels of endogenous HOCl. For plasma and supernatant samples, a 100 *μ*l sample was added to a 96-well microplate platform and incubated by 10 *μ*M HKOCl-3 for 30 min at 37°C. The fluorescence spectra were recorded by a microplate reader (BMG CLARIOstar, German) (excitation 494 nm; emission 555 nm).

### 2.5. Real-Time Quantitative PCR (qPCR)

Total RNA of cells was isolated and processed for qPCR using Applied Biosystems QuantStudio 5 (ThermoFisher). The following primers were used: (mouse) *Mpo* forward: GACATGCCCACCGAATGACAA, (mouse) *Mpo* reverse: CAGGCAACCAGCGTACAAAG; (human) *SOD1* forward: GTTGCAGTCCTCGGAACCAG, (human) *SOD1* reverse: CCACACCTTCACTGGTCCAT; (mouse) *SOD1* forward: TATGGGGACAATACACAAGGCT, (mouse) *SOD1* reverse: CGGGCCACCATGTTTCTTAGA; (mouse) *GAPDH* forward: TGACCTCAACTACATGGTCTACA, (mouse) *GAPDH* reverse: CTTCCCATTCTCGGCCTTG.

### 2.6. Western Blot

Total proteins of cells and tissues were isolated by RIPA buffer (Beyotime, P0013) contained with 1% phenylmethylsulfonyl fluoride (Beyotime, ST506), followed by separation on 12% SDS-PAGE gel. The following primary antibodies were used: hSOD1 (Abcam, ab52950), MPO (Abcam, ab188211), GPX4 (Affinity, DF6701), FSP1 (Affinity, DF8636), NQO1 (Affinity, DF6437), Bax (Beyotime, AB026), Bcl-2 (Affinity, BF9103), caspase-3 (Bioss, bs-0081R), LC3A/B (Cell Signaling Technology, 4108S), P62/SQSTM1 (Affinity, AF7875), and GAPDH (Cell Signaling Technology, 2118S). Goat anti-rabbit IgG (H+L) HRP (MultiSciences, 70-GAR0072) and goat anti-mouse IgG (H+L) HRP (MultiSciences, 70-GAM0072) were used as secondary antibodies. The densitometry of bands was quantified by ImageJ, and the bands of GAPDH served as the loading control.

### 2.7. Immunofluorescence

Mice were sacrificed by cardiac perfusion, and brains and spinal cords were fixed in 4% paraformaldehyde. After gradient dehydration, the tissues were then cryosectioned (brain: 10 *μ*m and lumbar spinal cords: 4 *μ*m) by LEICA CM1950. The frozen sections were heated and washed with PBS. Permeabilization was performed by 0.5% Triton X-100 and followed by blocking for 1 h. Cultured cells were washed with PBS and fixed with 4% PFA (paraformaldehyde). 0.3% Triton X-100 was utilized for permeabilization and blocked for 1 h at room temperature. The primary antibodies were incubated as follows: myeloperoxidase antibody (Abcam, ab9535, 1 : 100), MAP2 antibody (Proteintech, 17490-1-AP, 1 : 100), SMI-32 antibody (Abcam, ab8135, 1 : 1000), and FITC-anti-myeloperoxidase (Abcam, ab90812, 1 : 100). Alexa Fluor 555-labeled donkey anti-rabbit IgG (H+L) (Beyotime, A0453, 1 : 500) was used as the secondary antibody. The images were acquired with microscopes (Olympus BX63, Japan, and Axio Imager D2, Germany).

### 2.8. Nissl Staining

The morphology of motor neurons in brain and lumbar spinal cord sections was stained by Nissl staining solution (Beyotime, C0117) under the manufacturer's instructions. Briefly, the sections were fixed in 4% paraformaldehyde for 10 min and washed twice. After being stained by Nissl staining solution for 5-10 min at room temperature, the slices were dehydrated and cleared. The motor neurons in anterior horns of lumbar spinal cords were photographed with a microscope (Olympus BX63, Japan), and the brain sections were photographed by Axio Imager D2, Germany.

### 2.9. Measurements of Malondialdehyde (MDA)

Lipid peroxidation was assayed by the Lipid Peroxidation MDA Assay Kit (Beyotime, S0131S) according to the manufacturer's protocol. In brief, 10^6^ cells were homogenized in cold PBS, and after centrifugation, the supernatant was collected and quantified by the Pierce BCA Protein Assay Kit (ThermoFisher, 23227). 0.1 ml supernatant or plasma was mixed with 0.2 ml thiobarbituric acid (TBA) reagent. After boiling for 15 min, the mixture was cooled and centrifugated at 1000 g for 10 min. 200 *μ*l supernatant was measured photometrically by a microplate reader (BMG CLARIOstar, Germany) at 532 nm. The MDA levels were calculated by a standard curve, and the concentrations were expressed as nmol/mg protein for cells and *μ*mol/l for plasma.

### 2.10. Cell Death Assays

Cultured cells in 96-well plates were treated individually with vehicle, ferrostatin-1, Z-DEVD-FMK, or ABAH for 24 h or pretreated with NC-siRNA or MPO-siRNA as described in figure legends. The cell viability was analyzed by the cell viability assay (DOJINDO, CK04), and incubation with 10% CCK8 solution was performed at 37°C. The absorbance was determined by a microplate reader (BMG CLARIOstar, Germany).

For apoptosis detection, cells were treated with vehicle or ABAH for 24 h, and the Annexin V-PE/7-AAD Apoptosis Detection Kit (KeyGEN, KGA1018) was employed, and the ratio of apoptosis was measured by a flow cytometer (LSR II, BD).

### 2.11. Measurements of Myeloperoxidase (MPO) Activity

The MPO activity of plasma was measured by the Myeloperoxidase (MPO) Colorimetric Assay Kit (Elabscience, E-BC-K074-M-96T) according to the manufacturer's instructions. 45 *μ*l plasma per hole was added to the assay solution, and the change in absorbance was determined by a microplate reader (BMG CLARIOstar) at 460 nm. The MPO specific activity was calculated and expressed by units (U)/l plasma according to the formula: activity = Δ*A* × 0.175 × 1,000/*Vt*. Δ*A* is the change of absorbance, and *Vt* is the total volume.

### 2.12. Motor Performance

Mice were pretrained by rotarod testing for consecutive 7 days by rotarod apparatus (RotaRod Advanced, TSE) at a constant speed (5 rpm) for 5 min [29]. For detection, mice before sacrifice were placed on the rod at a gradually accelerated speed from 4 to 40 rpm. The test was performed twice each day with 30–40 min intervals, and the latency and speed to fall were obtained. The averaged time was recorded and analyzed in a blinded manner.

### 2.13. Statistical Analysis

Results were expressed as means ± SEM and obtained from at least three independent experiments. One-way ANOVA and Kruskal-Wallis test were used for multiple comparisons, while Student's *t*-test was employed for two datasets. The association between HOCl and ALSFRS-R scores was determined by Spearman's correlation coefficient. *P* < 0.05 was considered statistical significance.

## 3. Results

### 3.1. The Plasma Levels of HOCl Were Elevated in ALS Patients

**Figure 1:**
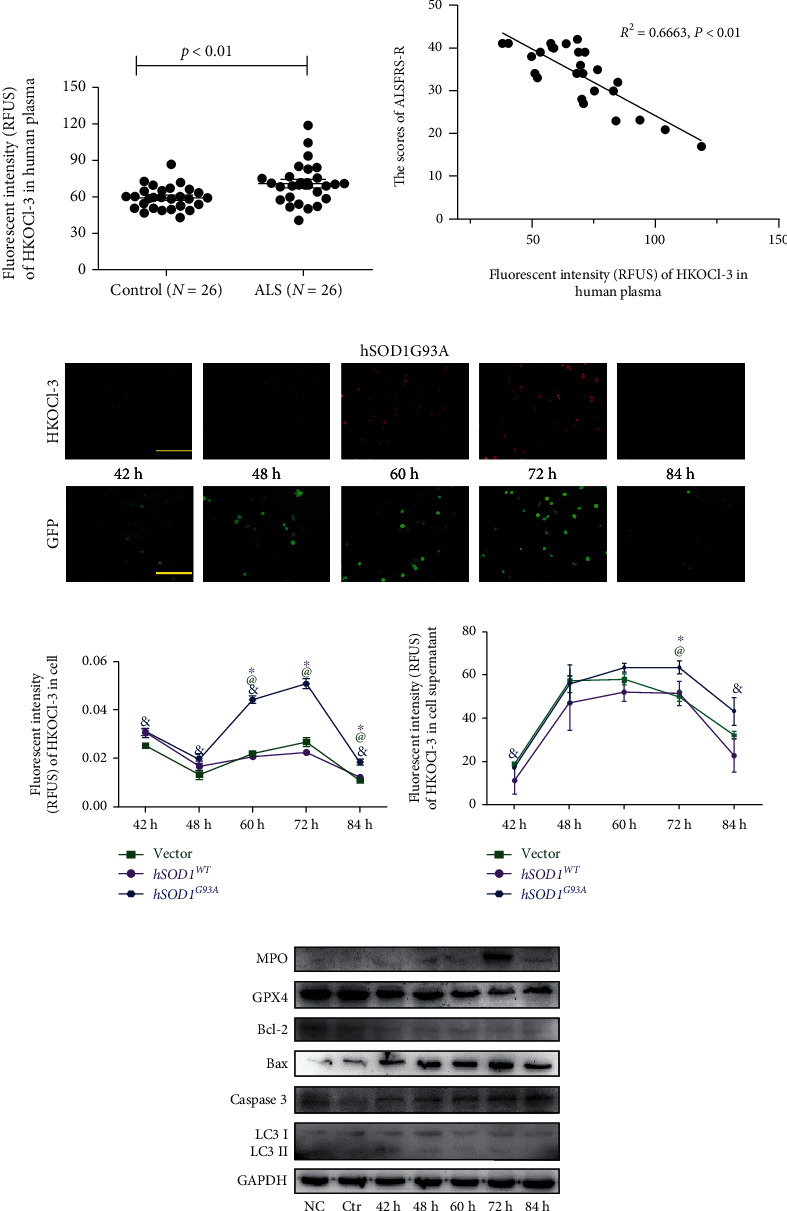
The associations between MPO/HOCl and hSOD1^G93A^ ALS. (a) The levels of plasma HOCl in healthy control and ALS patients. *P* < 0.01, Student's *t*-test. (b) The correction of plasma HOCl levels and ALSFRS-R scores in ALS patients (Spearman's rho = 0.6663, *P* < 0.01). (c) The time course analysis of endogenous HOCl and transfection efficiency in transiently transfected hSOD1^G93A^ NSC-34 cells. Bar = 200 *μ*m. (d, e) The quantitative analysis of fluorescent intensity of HKOCl-3 in cell and supernatant. ^&^*P* < 0.05 vs. 72 h in hSOD1^G93A^ NSC-34 cells, one-way ANOVA followed by LSD tests. ^@^*P* < 0.05 vs. vector, Student's *t*-test. ^∗^*P* < 0.05 vs. hSOD1^WT^. (f) The cell death of transiently transfected hSOD1^G93A^ NSC-34 cells, as measured by western blot.

Plasma samples were obtained from 26 controls and 26 patients with ALS. Complete data of ALSFRS-R scores were obtained from 26 patients. Compared to the control group, the fluorescent intensity of HKOCl-3 in plasma was remarkably increased in ALS patients (Figure 1(a)). The elevation of HOCl was negatively correlated with the scores of ALSFRS-R (Figure 1(b)), indicating the association of HOCl and pathologic progress of ALS.

### 3.2. Activation of MPO/HOCl Signaling in hSOD1^G93A^ NSC-34 Cell Models

Next, we built an ALS cell model by transfecting NSC-34 cells with plasmids harboring GFP-human SOD1 (wild-type (WT) or mutant (G93A)) as previously reported [7, 8]. Thirty-six h after transfection, the GFP-positive cells were sorted by flow cytometry and further cultured. At 42, 48, 60, 72, and 84 h after transfection, the endogenous and secreted HOCl were detected by the HKOCl-3 probe (Figure 1(c)). As the expression of GFP-hSOD1^G93A^ augmented, the levels of HOCl in the cells and supernatants both increased, peaking at 72 h and declining afterwards (Figures 1(d) and 1(e)). Consistently, the MPO protein expression also peaked at 72 h after transfection (Figure 1(f)). However, the expressions of MPO and HOCl remained constant in the vector and hSOD1^WT^ groups. We further confirmed that at 72 h, the *hSOD1* mRNA increased remarkably in the hSOD1^WT^ and hSOD1^G93A^ groups (S1A, left), while the transfection did not affect the expression of *mSOD1* (S1A, right), which was not correlated with ALS [30]. The results indicated that the upregulations of MPO and HOCl were due to the hSOD1^G93A^ mutation. To clarify the pathogenesis of MPO/HOCl in hSOD1^G93A^ NSC-34 cells, ferroptosis, apoptosis, and autophagy were detected, respectively, by the western blot assay (Figure 1(f), quantified analysis in S1B). The levels of GPX4 protein, an essential inhibitor of ferroptosis, decreased at 72 h after hSOD1^G93A^ transfection in NSC-34 cells. In addition, we observed the activation of caspase-3 and elevated ratios of Bax/Bcl-2, which are the features of apoptotic cell death. However, the conversion of LC3 I to LC3 II remained unchanged. The results demonstrated a possible crosslink between mutant hSOD1 protein and cellular ferroptosis and apoptosis, which is possibly mediated by MPO/HOCl.

### 3.3. Mutant hSOD1 Protein Mediated the Activation of MPO/HOCl

**Figure 2:**
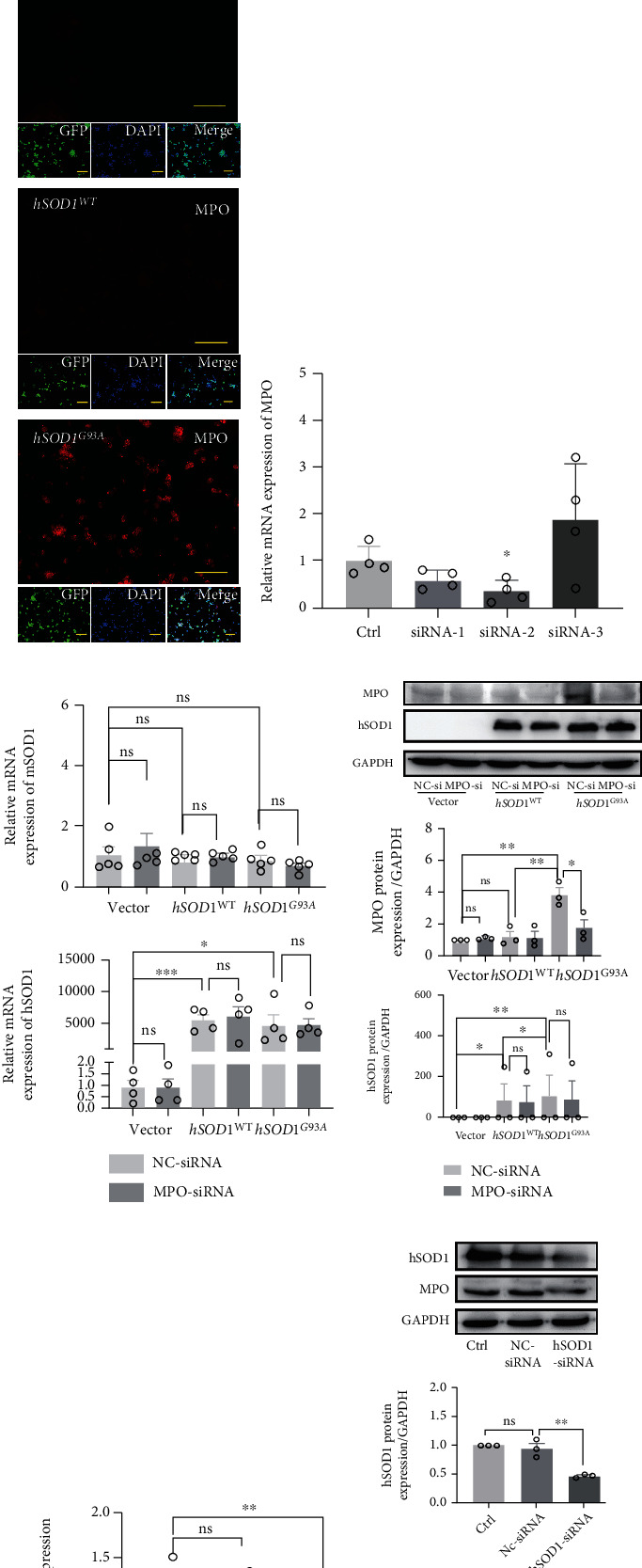
The mutant hSOD1 contributed to activation of MPO/HOCl. (a) The purity of vector, hSOD1^WT^, and hSOD1^G93A^ NSC-34 cells, as manifested by confocal images (Olympus FV10i, Japan). Bar = 40 *μ*m. (b) The *MPO* mRNA expression of vector, hSOD1^WT^, and hSOD1^G93A^ NSC-34 cells. (c) Representative immunofluorescence images of MPO in vector, hSOD1^WT^, and hSOD1^G93A^ NSC-34 cells. Bar = 200 *μ*m. (d) The *MPO* mRNA in hSOD1^G93A^ cells was downregulated significantly by MPO-siRNA-2. (e) The *mSOD1* (up) and *hSOD1* (down) mRNA expression in vector, hSOD1^WT^, and hSOD1^G93A^ NSC-34 cells treated with NC-siRNA or MPO-siRNA. (f) The hSOD1 expression in vector, hSOD1^WT^, and hSOD1^G93A^ NSC-34 cells treated with NC-siRNA or MPO-siRNA, as measured by western blot. (g) The *MPO* mRNA in hSOD1^G93A^ cells. (h) The MPO expression of hSOD1^G93A^ cells was downregulated by hSOD1 silencing. Quantified graphs were shown as means and SEM. ^∗^*P* < 0.05,  ^∗∗^*P* < 0.01, and^∗∗∗^*P* < 0.001, Student's *t*-test.

We subsequently generated NSC-34 cells that stably expressed GFP-human SOD1 (wild-type (WT) or mutant (G93A)). The purities of the GFP-positive cells were confirmed (Figure 2(a)), and MPO expression was detected by q-PCR (Figure 2(b)) and immunofluorescence staining (Figure 2(c)). Compared with the vector and hSOD1^WT^ groups, the mRNA (Figure 2(b)) and protein (Figure 2(c)) expressions of MPO were significantly increased in the hSOD1^G93A^ NSC-34 cells. Optimal MPO-siRNA was chosen by qPCR (Figure 2(d)). However, silencing MPO did not affect the expression of *mSOD1* mRNA (Figure 2(e), up) and *hSOD1* mRNAs (Figure 2(e), down) as well as proteins (Figure 2(f)). Subsequently, optimal hSOD1-siRNA was chosen by qPCR (S2C). The MPO mRNA and protein expressions in hSOD1^G93A^ NSC-34 cells were both inhibited by hSOD1 silencing (Figures 2(g) and 2(h)). The results suggested augmented mutant hSOD1 protein-induced upregulation of MPO and generation of HOCl, serving as the upstream activators.

### 3.4. MPO/HOCl Contributed to Apoptosis but Not Autophagy in hSOD1^G93A^ NSC-34 Motor Neurons

**Figure 3:**
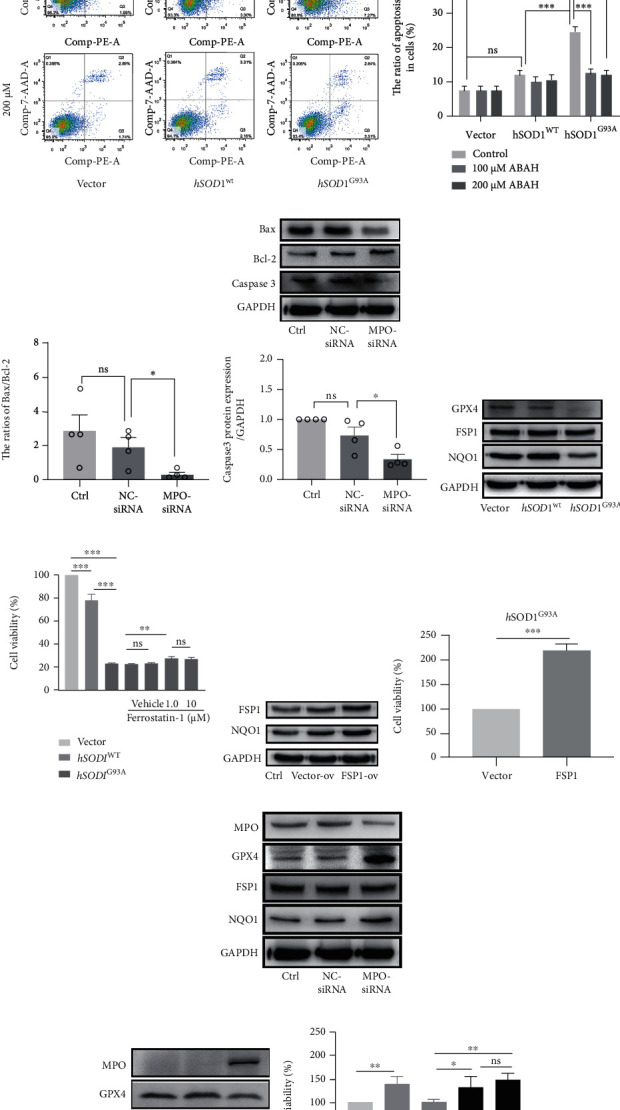
MPO/HOCl signaling manipulated apoptosis and ferroptosis in hSOD1^G93A^ cells. (a) The apoptosis- and autophagy-related proteins of vector, hSOD1^WT^, and hSOD1^G93A^ cells, as measured by western blot. (b) 1 *μ*M Z-DEVD-FMK reversed cell death of hSOD1^G93A^ cells, significantly. (c, d) Flow cytometry analysis of apoptosis in vector, hSOD1^WT^, and hSOD1^G93A^ cells, in the absence or presence of ABAH. (e) Immunoblots of hSOD1^G93A^ cells treated with NC-siRNA or MPO-siRNA. (f) The ferroptosis-related proteins of vector, hSOD1^WT^, and hSOD1^G93A^ cells, as measured by western blot. (g) 1 *μ*M ferrostatin-1 reversed the cell death in hSOD1^G93A^ cells, significantly. (h) Immunoblots of hSOD1^G93A^ overexpressed with vector or FSP1 cDNA. (i) Overexpression of FSP1 cDNA inhibited cell death significantly in hSOD1^G93A^ cells. (j) Immunoblots of ferroptosis-related proteins in hSOD1^G93A^ cells, treated with NC-siRNA or MPO-siRNA. (k) Immunoblots of hSOD1^G93A^ overexpressed with vector or MPO cDNA. (l) Relative viability of hSOD1^G93A^ cells with multiple treatments. (m) The MDA levels of cells in different groups. (n) Effects of different treatments on viability of NSC-34 cells overexpressed with empty vector or MPO cDNA. Quantified graphs were shown as means and SEM. ^∗^*P* < 0.05,  ^∗∗^*P* < 0.01, and^∗∗∗^*P* < 0.001. The significant difference among multiple doses was determined by one-way ANOVA followed by LSD tests. The significant difference in two datasets was analyzed by Student's *t*-test.

The caspase-3 expression and ratios of Bax/Bcl-2 were remarkably increased in hSOD1^G93A^ cells, indicating the activation of apoptosis (Figure 3(a), S2A). No difference was observed in the expression of the p62/SQSTM1 and conversion of LC3 I to LC3 II among the vector, hSOD1^WT^, and hSOD1^G93A^ groups, suggesting that hSOD1^G93A^ could not alter the autophagy pathway (Figure 3(a)). To further confirm the role of apoptosis in hSOD1^G93A^ cells, Z-DEVD-FMK, a caspase-3 inhibitor, was utilized and cell deaths in hSOD1^G93A^ cells were significantly decreased by 1.0 and 10.0 *μ*M Z-DEVD-FMK (Figure 3(b)). Our data suggested that apoptosis contributed to cell deaths in hSOD1^G93A^ cells. The apoptosis ratios were measured by an apoptosis detection kit (Figures 3(c) and 3(d)). The apoptosis ratios, at the early and late stages, were increased notably in hSOD1^G93A^ cells, which could be ameliorated by 4-aminobenzoic acid hydrazide (ABAH), an effective and selective MPO inhibitor. We silenced MPO expression with MPO-siRNA, which caused the caspase-3 upregulation and increased ratio of Bax/Bcl-2 to be reversed (Figure 3(e)).

### 3.5. MPO/HOCl Contributed to Ferroptosis in hSOD1^G93A^ NSC-34 Motor Neurons

To confirm our hypothesis that MPO/HOCl mediated ferroptosis in hSOD1^G93A^ NSC-34 cells, GPX4 and FSP1 (ferroptosis suppressor protein 1), a stand-alone parallel system from the glutathione-GPX4 axis, were detected by western blot [31].

The GPX4 protein decreased significantly in hSOD1^G93A^ cells (Figure 3(f), quantified analysis in S2B); however, FSP1 expression remained constant. NQO1 (NAD(P)H:Quinone Oxidoreductase 1), serving as a CoQ oxidoreductase, as FSP1 functions, supposedly cooperates in cellular resistance to ferroptosis in *Nature* [32] and was decreased significantly in hSOD1^G93A^ cells. To corroborate the existence of ferroptosis, ferrostatin-1 was added to suppress ferroptosis independently from apoptosis and necroptosis (Figure 3(g)). Cell death was tempered by 1.0 *μ*M of ferrostatin-1, suggesting that the glutathione-GPX4 axis mediated cell deaths in hSOD1^G93A^ cells. Similarly, FSP served as a ferroptosis suppressed pathway and accounted for ferroptosis resistance [32]. We therefore wondered whether FSP1 overexpression affected the cell death in hSOD1^G93A^ cells. We generated FSP1-overexpressed hSOD1^G93A^ cells and observed that the NQO1 expression was increased as FSP1s did (Figure 3(h), quantified analysis in S2C). FSP1 compensated for the inability of GPX4, by probably cooperating with NQO1. Remarkably, FSP1 overexpression increased cell viability in hSOD1^G93A^ cells (Figure 3(i)). Subsequently, the GPX4 and NQO1 expressions in hSOD1^G93A^ cells were rescued by MPO-siRNA (Figure 3(j), quantified analysis in S2D).

### 3.6. Inhibition of MPO/HOCl Ameliorated Lipid Peroxidation and Cell Death of hSOD1^G93A^ NSC-34 Motor Neurons

To further demonstrate the mediation of MPO/HOCl, we generated MPO-overexpressed NSC-34 cells. MPO overexpression inhibited GPX4 and NQO1 expression (Figure 3(k), quantified analysis in S2E). MPO silencing increased the cell viability of hSOD1^G93A^ NSC-34 cells (Figure 3(l)). The cell viabilities in hSOD1^G93A^ cells treated with Z-DEVD-FMK and ferrostatin-1 were similar. Lipid peroxidation was a signature of ferroptosis, and therefore, MDA assays were utilized (Figure 3(m)). Consistent with the results above, MPO overexpression increased MDA levels in NSC-34 cells, which might explain the increase of MDA in hSOD1^G93A^ cells compared to vector controls. Both MPO inhibitors and MPO-siRNAs could decrease the MDA levels in hSOD1^G93A^ cells. FSP1 overexpression also suppressed the MDA levels. Cell viability was decreased by MPO overexpression, supporting our hypothesis that MPO exerted cell deaths in hSOD1^G93A^ NSC-34 cells (Figure 3(n)). With MPO overexpression, no difference in cell death was observed in NSC-34 cells treated with Z-DEVD-FMK, ferrostatin-1, or ABAH. Activation of the MPO/HOCl pathway ultimately led to apoptosis and ferroptosis in hSOD1^G93A^ NSC-34 cells.

### 3.7. The MPO/HOCl Pathway Was Activated in hSOD1^G93A^ Mice

**Figure 4:**
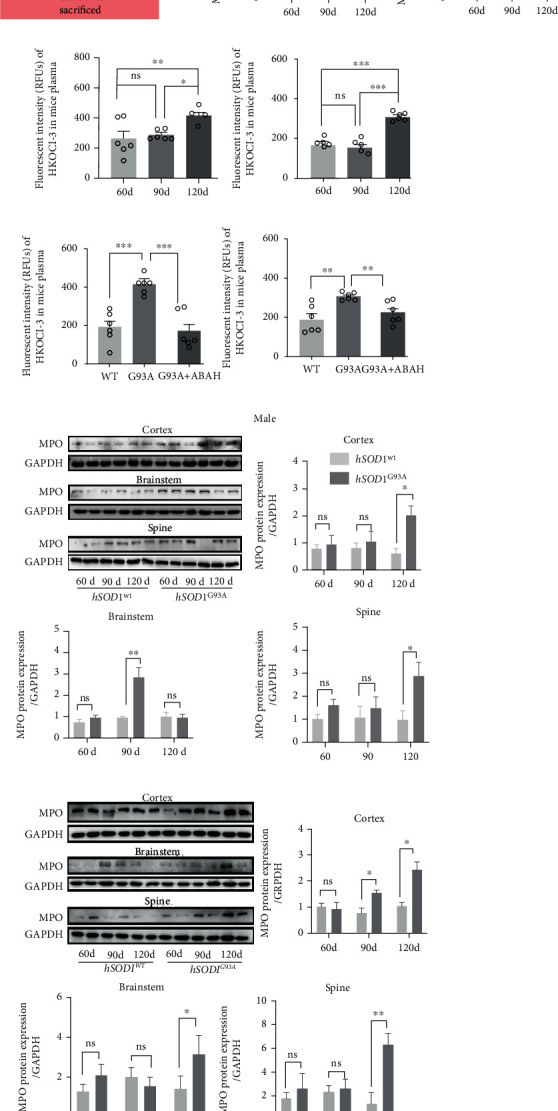
The activation of MPO/HOCl signaling in hSOD1^G93A^ mice. (a) Timeline of experimental design in mice. (b) The plasma MPO specific activity of hSOD1^G93A^ mice (left: male; right: female). (c) The plasma fluorescent intensity of HKOCl-3 in hSOD1^G93A^ mice (left: male; right: female). (d) The plasma fluorescent intensity of HKOCl-3 in hSOD1^WT^, hSOD1^G93A^, and hSOD1^G93A^ mice treated with ABAH at P120 (left: male; right: female). (e) Immunoblots of MPO in the motor cortex, brain stem, and spinal cord of male mice. (f) Immunoblots of MPO in the motor cortex, brain stem, and spinal cord of male mice. Quantified graphs were shown as means and SEM. ^∗^*P* < 0.05,  ^∗∗^*P* < 0.01, and^∗∗∗^*P* < 0.001. The significant differences in (b) and (c, male) were determined by the Kruskal-Wallis test. The significant differences in (c, female) were determined by one-way ANOVA followed by LSD tests. The significant difference in two datasets was analyzed by Student's *t*-test.

**Figure 5:**
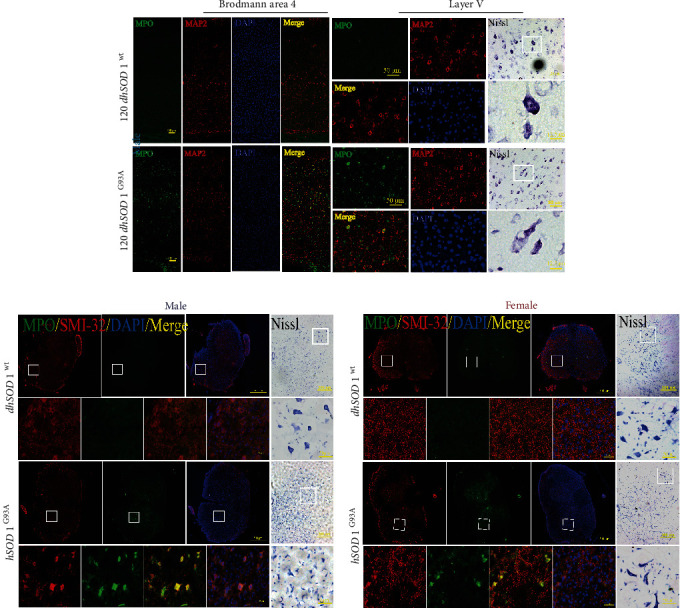
The cellular distribution of MPO-positive signals and Nissl staining in the motor cortex of male mice and spine of all mice. (a) The costaining of MPO (green), MAP2 (red), and DAPI (blue) at P120 in Brodmann area 4, bar = 100 *μ*m (zoom in layer V, bar = 50 *μ*m), and representative images of Nissl staining, bar = 50 *μ*m (zoom in, bar = 12.5 *μ*m). (b, c) The costaining of MPO (green), SMI-32 (red), and DAPI (blue) at P120 in lumbar ventral horn, bar = 500 *μ*m (zoom in, bar = 50 *μ*m), and representative images of Nissl staining, bar = 200 *μ*m (zoom in, bar = 50 *μ*m).

**Figure 6:**
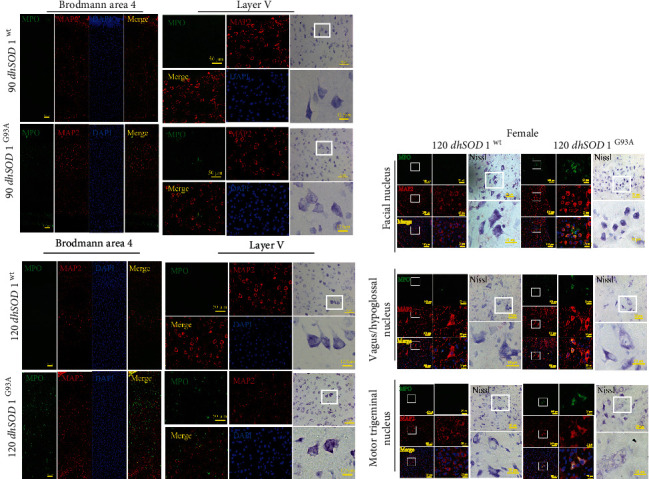
The cellular distribution of MPO-positive signals and Nissl staining in the motor cortex and brain stem of female mice. (a) The costaining of MPO (green), MAP2 (red), and DAPI (blue) at P90 and P120 in Brodmann area 4, bar = 100 *μ*m (zoom in layer V, bar = 50 *μ*m), and representative images of Nissl staining, bar = 50 *μ*m (zoom in, bar = 12.5 *μ*m). (b) The costaining of MPO (green), MAP2 (red), and DAPI (blue) in facial, vagus/hypoglossal, and motor trigeminal nucleus at P120, bar = 100 *μ*m (zoom in, bar = 25 *μ*m), and representative images of Nissl staining, bar = 20 *μ*m (zoom in, bar = 10 *μ*m).

MPO activity was detected in the plasma of the SOD1^G93A^ ALS mouse model (Figure 4(a)). The MPO activity of the male mice was increased significantly at postnatal day 90 (P90) and P120 (Figure 4(b)), while a significant increase of HOCl was observed at P120 (Figure 4(c)). In the female mice, the levels of MPO activity and HOCl increased remarkably at P120. At P120, the plasma HOCl levels of the male and female mice were decreased by ABAH (Figure 4(d)). To determine the regions where the MPO/HOCl pathway was activated, we detected the MPO expression in the motor cortices, brain stems, and spinal cords of the mice. Compared with SOD1^WT^ mice, a significant increase of MPO was observed in the motor cortex at P120 of the male SOD1^G93A^ mice (Figure 4(e)) while this increase was seen at P90 and P120 of the female mice (Figure 4(f)). Unexpectedly, the MPO expression increased at P90 in the brain stem of the male mice but returned to a basic level at P120, while the upregulation was observed at P120 in the brain stem of the female mice. The MPO expression increased significantly at P120 in the spines of both male and female mice. To confirm the cellular type, MPO-FITC antibodies were used and neurons were labeled by MAP2 in the mice's brains and SMI-32 in their spines. In Brodmann area 4 at P120, the MPO-positive cells were mainly located at pyramidal neurons in layers 3 and 5 in the male hSOD1^G93A^ mice (Figure 5(a)). Correspondingly, the neurons in layer 5 were degenerated in the hSOD1^G93A^ mice, as shown by Nissl staining. The brain stem motor nuclei, including facial, vagus/hypoglossal, and motor trigeminal nuclei, were detected. Although vacuolation and degeneration were observed in the brain stem motor nuclei of the hSOD1^G93A^ mice at P120 (S3), MPO-positive cells were hardly observed. At P120, the MPO-positive cells were observed in the lumbar ventral horn neurons of the male (Figure 5(b)) and female (Figure 5(c)) hSOD1^G93A^ mice, compared with hSOD1^WT^ mice. In addition, the numbers of motor neurons were decreased in the anterior horn of the hSOD1^G93A^ mice. Conversely, the weak MPO-positive cells were first observed at P90 in Brodmann area 4 of the female hSOD1^G93A^ mice (Figure 6(a)). However, MPO-positive cells were then observed in nearly all the layers of the motor cortex at P120. In layer 5, the MPO-positive cells were mainly in neurons, which were degenerated as Nissl staining showed. Also, the MPO-positive neurons were observed in the brain stem of female hSOD1^G93A^ mice at P120 (Figure 6(b)).

### 3.8. MPO/HOCl Regulated the Cell Death and Motor Performance in hSOD1^G93A^ Mice

**Figure 7:**
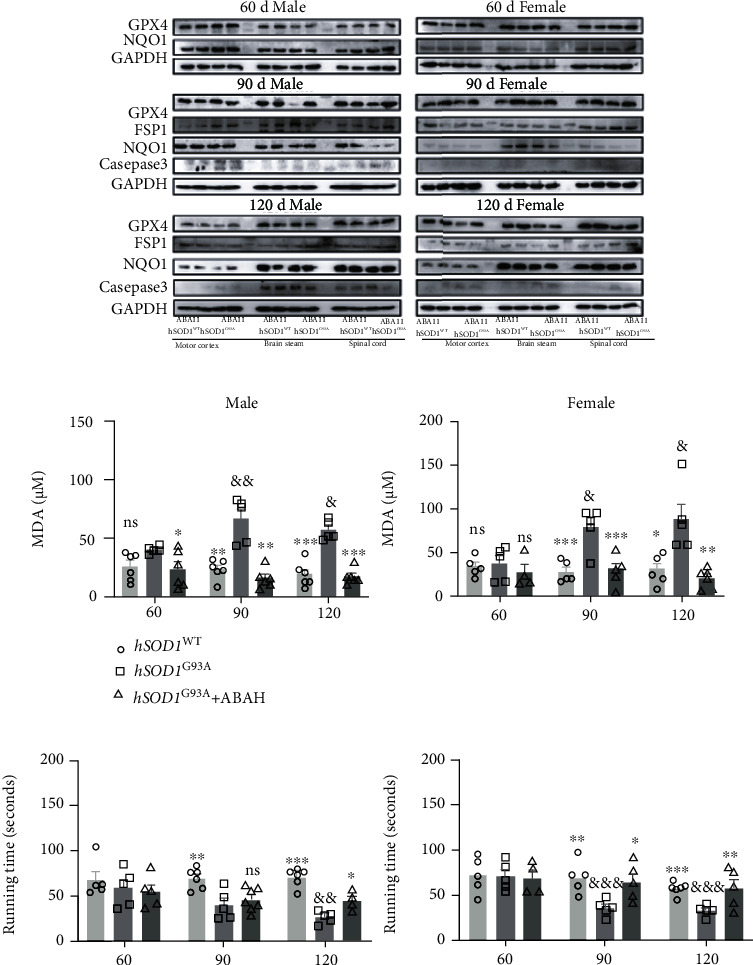
MPO/HOCl facilitated apoptosis and ferroptosis and exerted motor performance impaired in hSOD1^G93A^ mice. (a) Immunoblots of apoptosis- and ferroptosis-related proteins in hSOD1^WT^ and hSOD1^G93A^ mice, with or without treatment of ABAH in male (left) and female (right) mice. (b) The plasma MDA levels of male (left) and female (right) mice. (c) The motor performance of mice (left: male; right: female). Quantified graphs were shown as means and SEM. ^∗^*P* < 0.05,  ^∗∗^*P* < 0.01, and^∗∗∗^*P* < 0.001. The significant difference in two datasets was analyzed by Student's *t*-test.

To determine the cell death at different stages of hSOD1^G93A^ mice, we performed western blot in their motor cortex, brain stem, and spinal cord (Figure 7(a), quantified analysis in S4A-C). GPX4 and NQO1 protein levels were decreased in the brain stem at P90 in the male hSOD1^G93A^ mice (Figure 7(a), S4B), which is consistent with the results above. At P120, GPX4 decreased in the motor cortex and spine while NQO1 only decreased in the cortex in the male hSOD1^G93A^ mice (Figure 7(a), S4C). In addition, caspase-3 expression increased at the motor cortex, brain stem, and spine at both P90 and P120. The changes, except for the brain stem at P120, were reversed by ABAH treatment for 7 days.

GPX4 protein levels decreased in the motor cortex at P90 and P120, while NQO1 decreased in the motor cortex at P120 of the female hSOD1^G93A^ mice, similar to the male mice. However, the caspase-3 expression increased in all three regions at P120 of the female mice. As expected, ABAH treatment inhibited the changes in the female mice. Subsequently, the plasma MDA level was detected, and significant differences were observed in the hSOD1^G93A^ mice at P90 and P120 (Figure 7(b)), which was reversed by ABAH treatment.

The results suggested that ferroptosis occurred at the early stage and late stage of the disease and could be regulated by MPO activity. Apoptosis accounted for cell deaths at P90 and P120 for the male mice while at P120 for the female mice. The mice's motor performance was further measured by a rotarod test, and significant impairment was observed at P90 and P120 in the hSOD1^G93A^ mice (Figure 7(c)), compared to the hSOD1^WT^ mice. Improvement in behavior was observed at P120 in ABAH-treated hSOD1^G93A^ male mice and at P90 and P120 in female mice.

**Figure 8:**
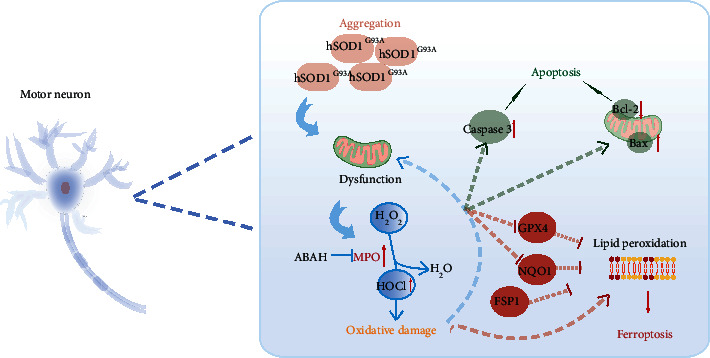
Graphic summary. Aggregation of mutant hSOD1 protein impaired mitochondria, contributing to activation of MPO/HOCl in hSOD1^G93A^ ALS. MPO/HOCl facilitated apoptosis and ferroptosis in motor neurons through upregulating the expression of caspase-3 and Bax/Bcl-2 or inhibiting Gpx4 and NQO1. Inhibition of Gpx4 and NQO1 contributed to lipid peroxidation, the signature of ferroptosis, which could be alleviated by FSP1.

The MPO/HOCl pathway was activated in the disease progress of the hSOD1^G93A^ ALS mice and might cause cell deaths in different regions at various stages of disease (Figure 8), thus manipulating the mice's motor performance.

## 4. Discussion

We investigated a novel potential mechanism to explain progressive motor neuronal deaths in hSOD1^G93A^ ALS. Our study focused on the accumulation of HOCl in ALS patients and the activation of the MPO/HOCl pathway in hSOD1^G93A^ motor neuron cells and hSOD1^G93A^ transgenic mice. MPO/HOCl signaling contributed to apoptosis and ferroptosis in the neurons of hSOD1^G93A^ cells and mouse models, ultimately affecting the motor performance in mice.

MPO is a heme-containing peroxidase and is expressed in neutrophils, activated microglia, astrocytes, neurons, etc., contributing to the neuropathology of multiple neurodegenerative diseases [33]. Enzymatically active MPO catalyzes the conversion of H_2_O_2_ and Cl^−^ to HOCl, which is a powerful oxidant and exerts oxidative damage [34]. Furthermore, the activity of plasma MPO is a supposed biomarker of several cardiac scenarios and neurodegenerative disorders like Alzheimer's disease [35]. Researchers' attention has been drawn to the cytotoxicity of MPO. Research has seldom delineated the function of HOCl, the direct actor in oxidative stress, due to inadequate detection methods. HKOCl-3, a selective and sensitive fluorescent HOCl probe, provided an innovative investigation for unraveling the role of HOCl [28]. Using the HKOCl-3 probe, we discovered increased levels of plasma HOCl in ALS patients, which was correlated with the ALSFRS-R scores, predicting the degree of neurological deficits. Then, we monitored the activation of the MPO/HOCl pathway with misfolded hSOD1^G93A^ proteins aggregating in a motor neuron cell model transiently expressing hSOD^G93A^ genes. The activation degree of the MPO/HOCl pathway in hSOD1^G93A^ transgenic NSC-34 cells was associated with the transfection efficacy of GFP*-hSOD1^G93A^*, suggesting a crosslink of aggregated hSOD1^G93A^ proteins and MPO/HOCl. On the other hand, the expressions of hSOD1 mRNAs and proteins were unaffected by MPO silencing while downregulation of MPO was observed following hSOD-siRNA transfection in NSC-34 cells. This underscored the notion that aggregation of misfolded hSOD1^G93A^ proteins triggered activation of the MPO/HOCl pathway and induced oxidative stress. However, the disease-modulatory mechanisms of MPO/HOCl in the neuropathology of ALS remained elusive.

Ferroptosis, a nonapoptotic form of cell death, was reported to be a combination of iron-dependent and accumulated lipid peroxidation products [36]. The imbalance of the generation of ROS and elimination of lipid peroxidation contributed to triggering and accelerating ferroptosis. Glutathione peroxidase 4 (GPX4), cooperating with glutathione (GSH), promoted the reduction of lipid peroxides and prevented cellular and membranous peroxidation, ultimately suppressing ferroptosis [37]. GPX4, serving as the crossroads of lipid peroxidation and ferroptosis, has been recognized as a critical mediator in ferroptosis, and GPX4 inhibitors were generated to induce ferroptosis. Recently, ferroptosis suppressor protein 1 (FSP1), was demonstrated to catalyze the production of coenzyme Q10 using cosubstrate NAD(P)H, facilitating ferroptosis resistance in parallel with the GPX4-GSH axis [31]. NQO1 functioned as a CoQ oxidoreductase and mitochondrial ROS inhibitor and has been demonstrated to suppress ferroptosis [38]. A lack of FSP1 and NQO1 deteriorated the cells' susceptibility to ferroptosis, compared with FSP1^KO^ cells [32]. Thus, companions of FSP and NQO1 might account for the ferroptosis resistance independently from the glutathione-dependent GPX4 signaling pathway. In our study, we discovered that the expression of GPX4 was remarkably inhibited in hSOD1^G93A^ NSC-34 cells, correlated with the accumulated hSOD1^G93A^ proteins and activation of the MPO/HOCl pathway. Although FSP1 expression remained unaffected, NQO1 expression was significantly inhibited and overexpression of FSP1 enabled the cell viability and MDA levels recovered in hSOD1^G93A^ cells. We speculated that FSP and NQO1 might compensate for the loss of GPX4 activity, and the two parallel systems were both interrupted in the hSOD1^G93A^ ALS model. To further verify the role of MPO/HOCl in cell deaths of the hSOD1^G93A^ ALS model in *vitro*, we silenced the MPO expression in hSOD1^G93A^ NSC-34 cells or overexpressed MPO in NSC-34 cells. Our data supported the hypothesis that overactivation of the MPO/HOCl pathway triggered lipid peroxidation and cell deaths, including apoptosis and ferroptosis in hSOD1^G93A^ NSC-34 cells. Balance of power between apoptosis and ferroptosis might account for the similar effect of ferrostatin-1 and Z-DEVD-FMK on cell viability.

*In vivo*, the distribution of enhanced MPO was analyzed. MPO expression was increased in brain stems at P90 while at P120 in the cortices and spinal cords of male mice. Previous studies demonstrated that degeneration occurred in most motor nuclei of brain stems and mutant SOD1 aggregated in the remnant motor nuclei of mice [39, 40]. We therefore supposed that the motor nuclei degenerated remarkably at P120 in male mice and caused apoptosis and cell deaths, lacking the ability to express more mutant SOD1 and MPO. At P90, loss of GPX4 proteins occurred not only in brain stems but also in spines, which was reversed by ABAH treatment. The inconsequent results compared with MPO expression suggested that MPO activity might be enhanced prior to MPO expression in the spines of male mice. On the other hand, whether other mechanisms facilitated ferroptosis in hSOD1^G93A^ ALS mice requires further elucidation. Apoptosis accounted for the cell deaths in the motor cortices, brain stems, and spines at P90 and P120 in the male mice, while the MPO expression was increased in the mice's brain stems at P90 and cortices and spinal cords at P120. We speculated that apoptosis experimentally happened beginning at the early stage of hSOD1^G93A^ male mice and MPO/HOCl could partly account for the apoptotic cell deaths. Thus, inhibiting MPO was not sufficient to ameliorate motor performance at P90 in the male mice. The significant increase of plasma HOCl as well as apoptosis and ferroptosis in mice's brain was inhibited at P120 in hSOD1^G93A^ male mice, which might account for the ameliorated motor deficit by ABAH treatment at P120 of male mice.

The loss of GPX4 occurred in regions exactly where MPO expression was increased in the female mice at P90 and P120. Paralleling to the brain, the plasma MDA levels were increased significantly at P90 and P120, which could be inhibited by ABAH treatment, underlying a prominent role of MPO/HOCl in facilitating ferroptosis in hSOD1^G93A^ female mice. Ferroptosis occurred prior to apoptosis, which were both suppressed significantly by ABAH. Thus, ABAH treatment was able to temper neurological deficits at P90 and P120 in the female mice. In addition, enhanced MPO expression occurred in more regions of the cortex and brain stem in female mice at P120, compared to male mice. Herein, we hypothesized that treatment targeting at MPO/HOCl could improve motor function of hSOD1^G93A^ ALS, especially in female patients. There is evidence that ALS has sexual differences including incidence, age of onset, and risk of executive dysfunction [41, 42]. It is difficult to explain the sexual specific differences by a single mechanism. Possible reasons for the differences in ALS between men and women include different environmental exposures, different biological responses to exogenous toxins such as a possible neuroprotective and neurotrophic effect of steroid hormones [43], and possibly different repair capacity of nervous systems [44]. However, further investigations are still required to uncover the mechanisms.

Counterintuitively, MPO expression was upregulated in a cellular-specific manner. Motor neurons were characterized as the prominent location where MPO/HOCl contributed to cell deaths in hSOD1^G93A^ mice. In the motor cortex, MPO-positive cells were prominent in the cortical pyramidal neurons, consistent with previous studies that oxidative damage occurred mainly in layers III-V pyramidal neurons of postmortem tissues in ALS patients [45]. Motor neurons were more vulnerable to cytotoxicity and degeneration, making them more susceptible to oxidative damage [46]. However, the mechanisms on why MPO expression was enhanced mostly in motor neurons require further study.

## 5. Conclusion

In conclusion, we discovered that MPO/HOCl facilitated apoptosis and ferroptosis in the motor neurons of the hSOD1^G93A^ ALS mouse model in an integrative way, ultimately leading to neurological deficits. Targeting MPO/HOCl signaling might be an effective therapy for motor performance improvement in hSOD1^G93A^ mutation-related ALS patients, especially female patients.

## Data Availability

All supporting data for this manuscript are included in the figures and supplementary materials. They are also available on reasonable request, and the corresponding author should be contacted to request the data.

## Abbreviations

MPO: Myeloperoxidase
HOCl: Hypochlorous acid
ROS: Reactive oxygen species
SOD1: Cu/Zn-superoxide dismutase
ALS: Amyotrophic lateral sclerosis
GPX4: Glutathione peroxidase 4
NQO1: NAD(P)H:Quinone Oxidoreductase 1
FSP1: Ferroptosis suppressor protein 1
ABAH: 4-Aminobenzoic acid hydrazide
MDA: Malondialdehyde.

## Supplementary Materials

Supplementary 1 S1: additional results for transient cell transfection and selection of optimal hSOD1-siRNA. (A) The *hSOD1* mRNA and *mSOD1* mRNA expression in cells at 72 h after transfection. (B) The quantified analysis of immunoblots in Figure 1(f). (C) The hSOD1-siRNA-3 was chosen for hSOD1 silencing. ^∗^*P* < 0.05,  ^∗∗^*P* < 0.01, and^∗∗∗^*P* < 0.001. The significant difference in two datasets was analyzed by Student's *t*-test. The significant difference among different times after transfection (Ctr, 42 h, 48 h, 60 h, 72 h, and 84 h) was determined by one-way ANOVA followed by LSD tests.

Supplementary 2 S2: the quantified analysis of immunoblots in Figure 3. (A) The quantified analysis of immunoblots in Figure 3(a). (B) The quantified analysis of immunoblots in Figure 3(f). (C) The quantified analysis of immunoblots in Figure 3(h). (D) The quantified analysis of immunoblots in Figure 3(j). (E) The quantified analysis of immunoblots in Figure 3(k). ^∗^*P* < 0.05,  ^∗∗^*P* < 0.01, and^∗∗∗^*P* < 0.001. The significant difference in two datasets was analyzed by Student's *t*-test.

Supplementary 3 S3: the cellular distribution of MPO-positive signals and Nissl staining in brain stem of male mice. (A) The costaining of MPO (green), MAP2 (red), and DAPI (blue) in facial, vagus/hypoglossal, and motor trigeminal nucleus at P120, bar = 100 *μ*m (zoom in, bar = 25 *μ*m), and representative images of Nissl staining, bar = 20 *μ*m (zoom in, bar = 10 *μ*m).

Supplementary 4 S4: the quantified analysis of immunoblots in Figure 7. (A) The quantified analysis of immunoblots of mice at P60 in Figure 7(a). (B) The quantified analysis of immunoblots of mice at P90 in Figure 7(a). (C) The quantified analysis of immunoblots of mice at P120 in Figure 7(a). ^∗^*P* < 0.05,  ^∗∗^*P* < 0.01, and^∗∗∗^*P* < 0.001. The significant difference in two datasets was analyzed by Student's *t*-test.
