# Supplementary figures and images for: MPO/HOCl Facilitates Apoptosis and Ferroptosis in the SOD1G93A Motor Neuron of Amyotrophic Lateral Sclerosis
Source: Oxid Med Cell Longev. 2022 Feb 7;2022:8217663. doi: 10.1155/2022/8217663 (PMC8845144; doi:10.1155/2022/8217663)

A

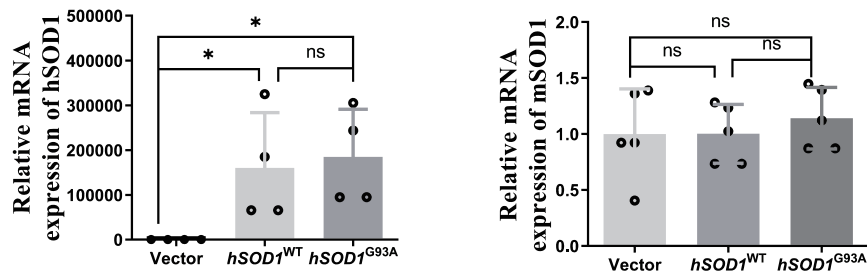

B

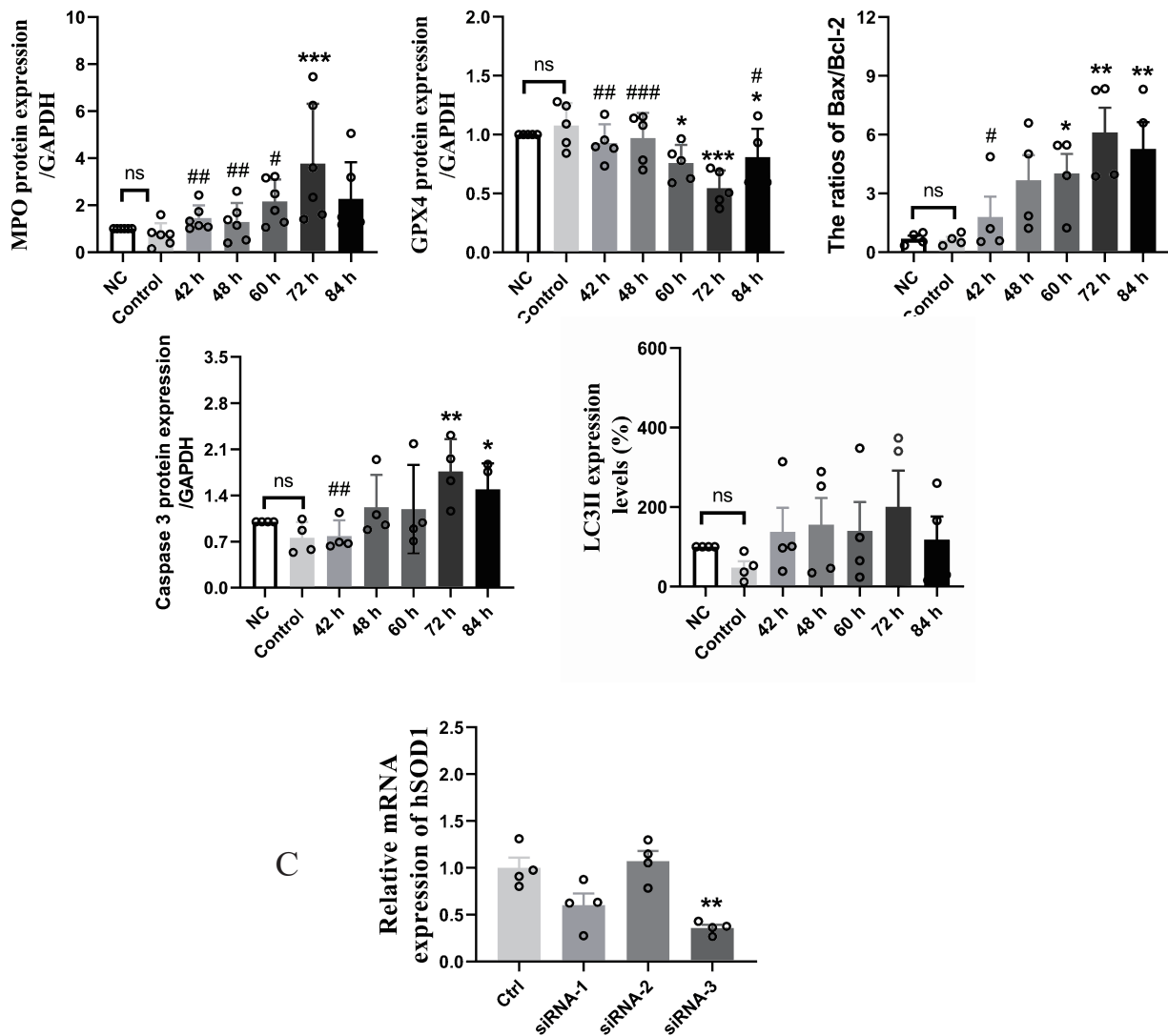

Supplement: Supplementary 1 — S1: additional results for transient cell transfection and selection of optimal hSOD1-siRNA. (A) The hSOD1 mRNA and mSOD1 mRNA expression in cells at 72 h after transfection. (B) The quantified analysis of immunoblots in Figure 1(f). (C) The hSOD1-siRNA-3 was chosen for hSOD1 silencing. ∗P < 0.05, ∗∗P < 0.01, and∗∗∗P < 0.001. The significant difference in two datasets was analyzed by Student's t-test. The significant difference among different times after transfection (Ctr, 42 h, 48 h, 60 h, 72 h, and 84 h) was determined by one-way ANOVA followed by LSD tests. [file 8217663.f1.pdf]

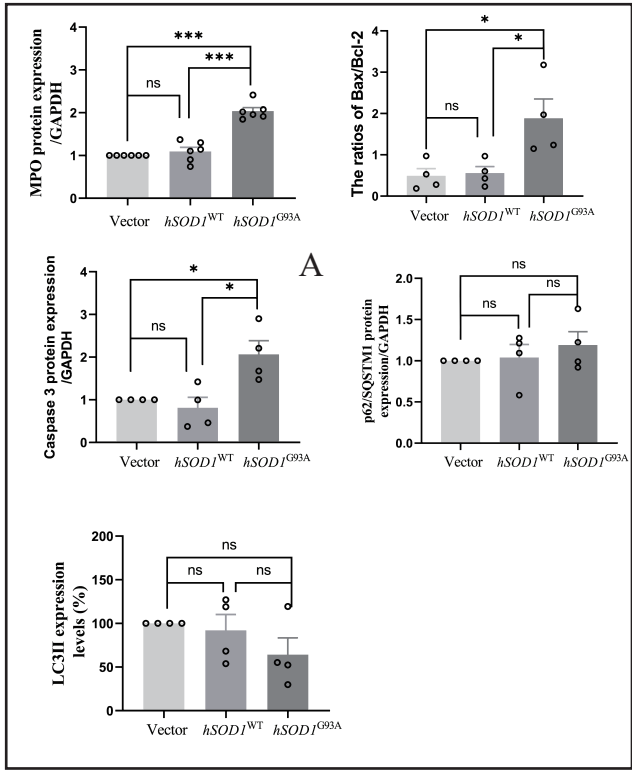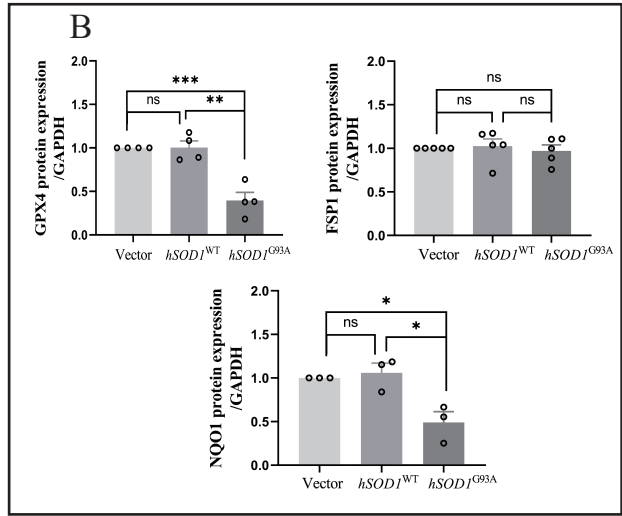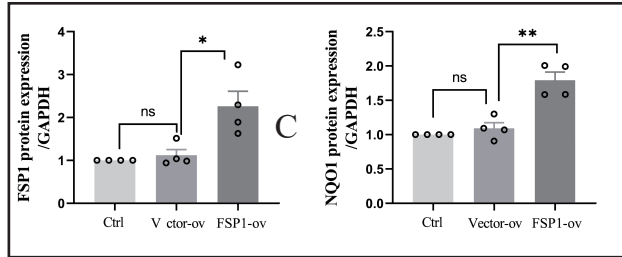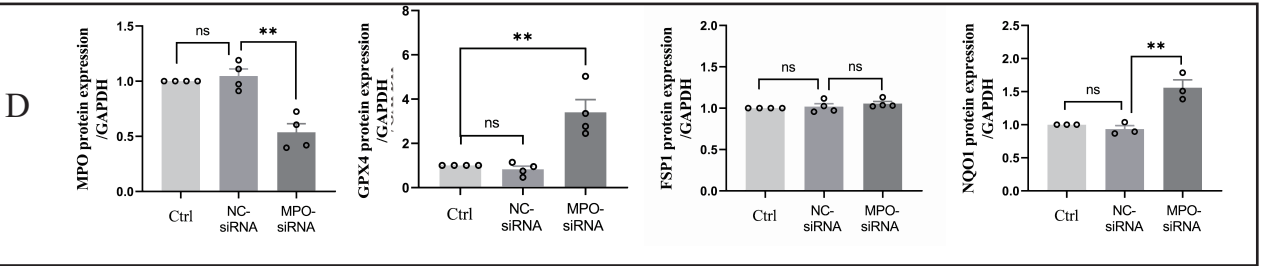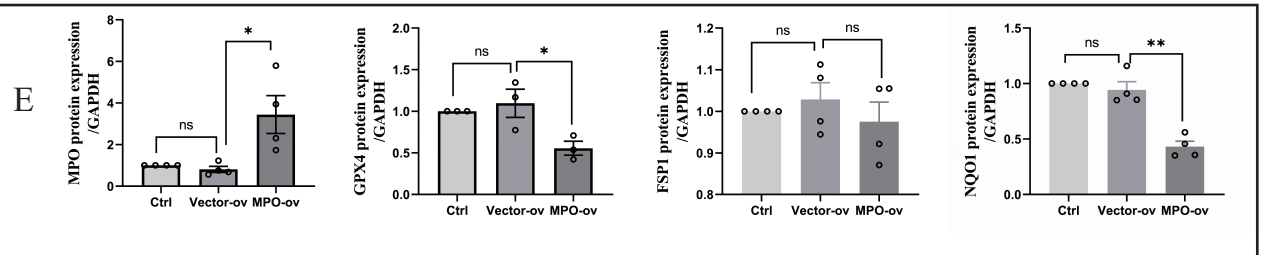

Supplement: Supplementary 2 — S2: the quantified analysis of immunoblots in Figure 3. (A) The quantified analysis of immunoblots in Figure 3(a). (B) The quantified analysis of immunoblots in Figure 3(f). (C) The quantified analysis of immunoblots in Figure 3(h). (D) The quantified analysis of immunoblots in Figure 3(j). (E) The quantified analysis of immunoblots in Figure 3(k). ∗P < 0.05, ∗∗P < 0.01, and∗∗∗P < 0.001. The significant difference in two datasets was analyzed by Student's t-test. [file 8217663.f2.pdf]

Male

Female

A

60 d

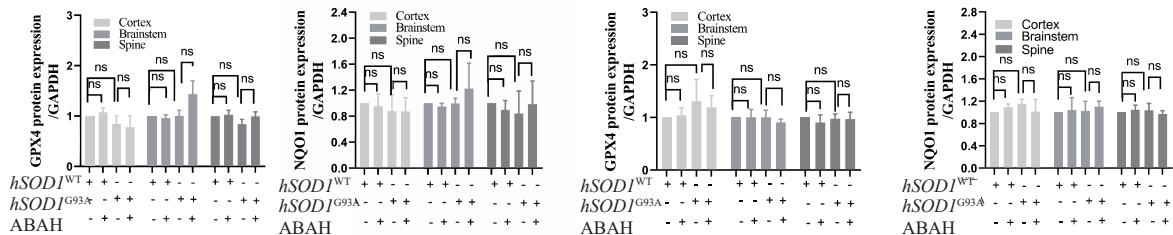

B 90 d

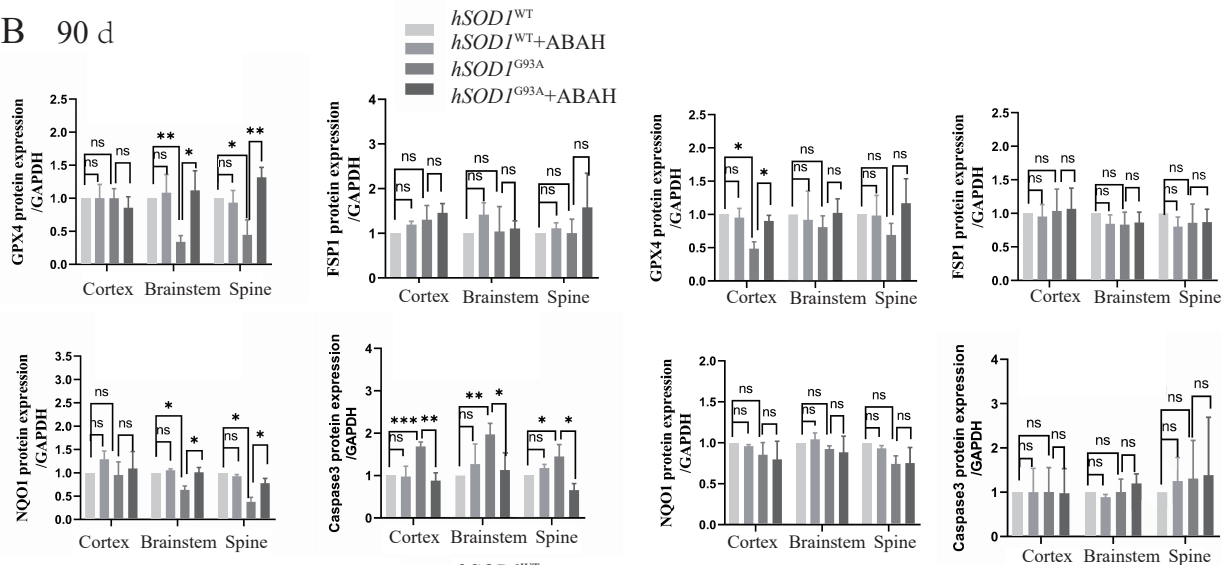

C 120 d

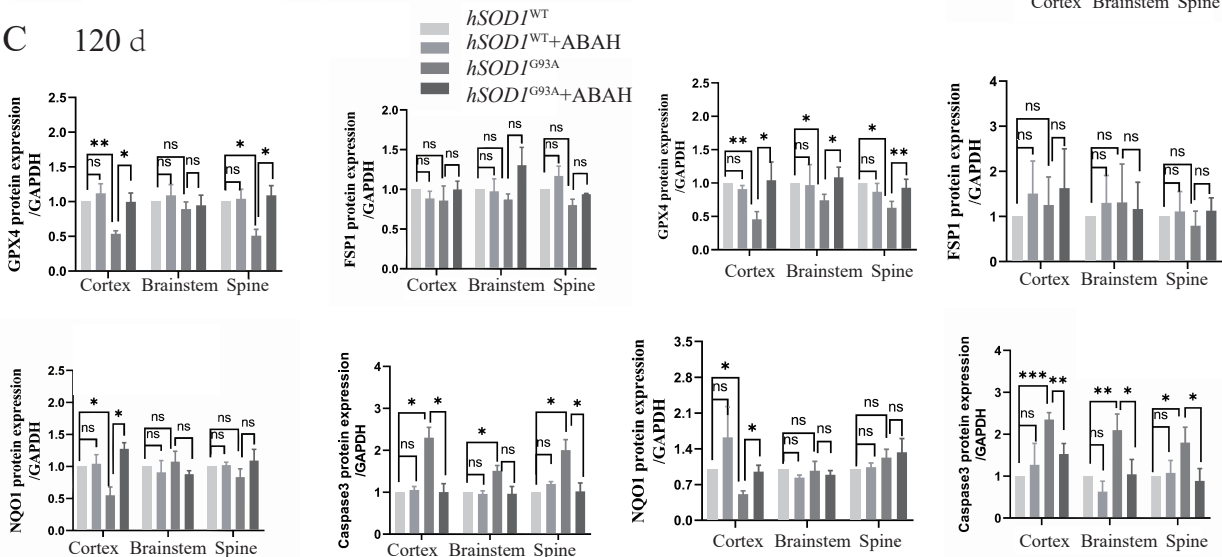

Supplement: Supplementary 4 — S4: the quantified analysis of immunoblots in Figure 7. (A) The quantified analysis of immunoblots of mice at P60 in Figure 7(a). (B) The quantified analysis of immunoblots of mice at P90 in Figure 7(a). (C) The quantified analysis of immunoblots of mice at P120 in Figure 7(a). ∗P < 0.05, ∗∗P < 0.01, and∗∗∗P < 0.001. The significant difference in two datasets was analyzed by Student's t-test. [file 8217663.f4.pdf]
