# Supplementary material for: MPO/HOCl Facilitates Apoptosis and Ferroptosis in the SOD1G93A Motor Neuron of Amyotrophic Lateral Sclerosis
Source: Oxid Med Cell Longev. 2022 Feb 7;2022:8217663. doi: 10.1155/2022/8217663 (PMC8845144; doi:10.1155/2022/8217663)
Supplement: Supplementary 3 — S3: the cellular distribution of MPO-positive signals and Nissl staining in brain stem of male mice. (A) The costaining of MPO (green), MAP2 (red), and DAPI (blue) in facial, vagus/hypoglossal, and motor trigeminal nucleus at P120, bar = 100 μm (zoom in, bar = 25 μm), and representative images of Nissl staining, bar = 20 μm (zoom in, bar = 10 μm). [file 8217663.f3.pdf]

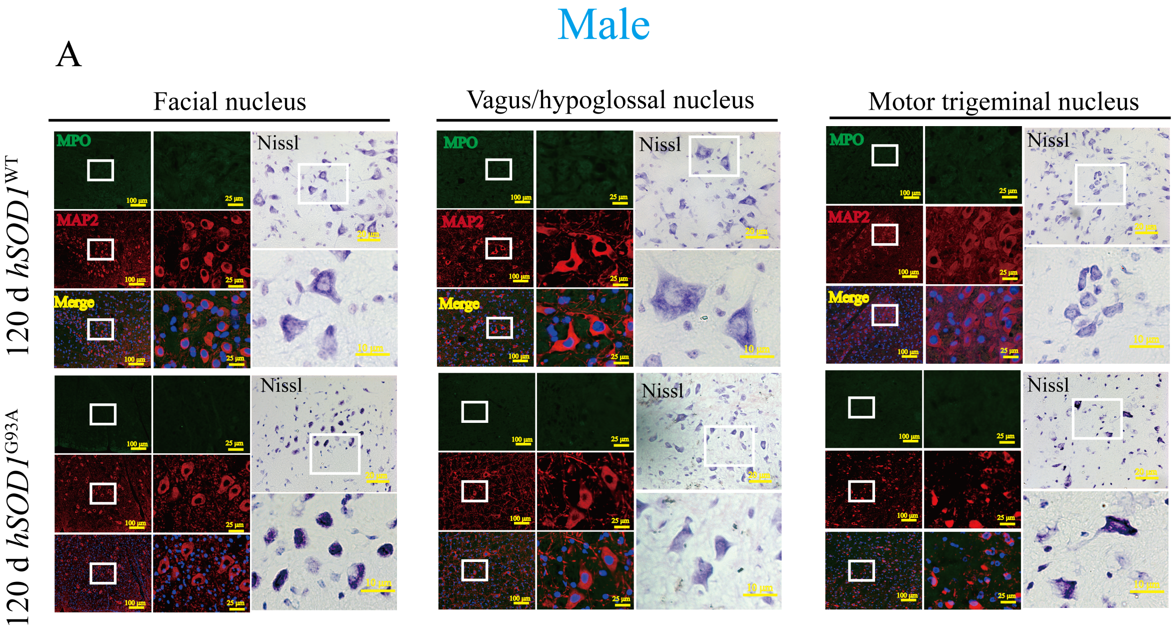
Male
A
Facial nucleus
Vagus/hypoglossal nucleus
Motor trigeminal nucleus
120 d hSOD1WT
120 d hSOD1G93A
MPO
MAP2
Merge
Nissl
100 µm
25 µm
20 µm
10 µm
